# Performance of artificial intelligence in 7533 consecutive prevalent screening mammograms from the BreastScreen Australia program

**DOI:** 10.1007/s00330-023-10396-7

**Published:** 2023-11-13

**Authors:** John Waugh, Jill Evans, Miranda Miocevic, Darren Lockie, Parisa Aminzadeh, Anne Lynch, Robin J. Bell

**Affiliations:** 1 Monash BreastScreen, Monash Cancer Centre, Moorabbin Hospital, 823-865 Centre Road, Bentleigh East, Victoria 3165 Australia; 2 Women’s Health Research Program, School of Public Health and Preventive Medicine, Monash University, Melbourne, Victoria 3004 Australia

**Keywords:** Mammography, Artificial intelligence, Breast cancer, Cancer screening

## Abstract

**Objectives:**

To assess the performance of an artificial intelligence (AI) algorithm in the Australian mammography screening program which routinely uses two independent readers with arbitration of discordant results.

**Methods:**

A total of 7533 prevalent round mammograms from 2017 were available for analysis. The AI program classified mammograms into deciles on the basis of breast cancer (BC) risk. BC diagnoses, including invasive BC (IBC) and ductal carcinoma in situ (DCIS), included those from the prevalent round, interval cancers, and cancers identified in the subsequent screening round two years later. Performance was assessed by sensitivity, specificity, positive and negative predictive values, and the proportion of women recalled by the radiologists and identified as higher risk by AI.

**Results:**

Radiologists identified 54 women with IBC and 13 with DCIS with a recall rate of 9.7%. In contrast, 51 of 54 of the IBCs and 12/13 cases of DCIS were within the higher AI score group (score 10), a recall equivalent of 10.6% (a difference of 0.9% (CI −0.03 to 1.89%, *p *= 0.06). When IBCs were identified in the 2017 round, interval cancers classified as false negatives or with minimal signs in 2017, and cancers from the 2019 round were combined, the radiologists identified 54/67 and 59/67 were in the highest risk AI category (sensitivity 80.6% and 88.06 % respectively, a difference that was not different statistically).

**Conclusions:**

As the performance of AI was comparable to that of expert radiologists, future AI roles in screening could include replacing one reader and supporting arbitration, reducing workload and false positive results.

**Clinical relevance statement:**

AI analysis of consecutive prevalent screening mammograms from the Australian BreastScreen program demonstrated the algorithm’s ability to match the cancer detection of experienced radiologists, additionally identifying five interval cancers (false negatives), and the majority of the false positive recalls.

**Key Points:**

*• The AI program was almost as sensitive as the radiologists in terms of identifying prevalent lesions (51/54 for invasive breast cancer, 63/67 when including ductal carcinoma in situ).*

*• If selected interval cancers and cancers identified in the subsequent screening round were included, the AI program identified more cancers than the radiologists (59/67 compared with 54/67, sensitivity 88.06 % and 80.6% respectively p = 0.24).*

*• The high negative predictive value of a score of 1–9 would indicate a role for AI as a triage tool to reduce the recall rate (specifically false positives).*

## Introduction

Desirable innovations in mammographic screening include reducing false positive recalls while maintaining sensitivity, as well as minimising interval cancers. An innovation involving AI could have the added advantage of reducing workload for radiologists, who are in short supply [1, 2].

Evaluation of AI in screening mammography is fraught for a number of reasons. A recent systematic review [3] indicated that only 5 of 36 AI systems performed better than a single radiologist reader, and that none exceeded the performance of a dual reader system with arbitration. However, few studies have data on interval cancers following the index screening round or data on cancers detected at the subsequent screening round. Assessment of AI has also frequently occurred in a “research environment” using highly enriched data sets as opposed to in a “work as usual” environment using an unselected population where the prevalence of true positives is typically < 1.2% [4].

The aims of our study were to investigate the performance of an established AI product in women undergoing their prevalent full-field digital screening mammogram during one calendar year at one BreastScreen Australia (BSA) service, including lesions identified during the prevalent screen, interval cancers, and next round (verified 3-year follow-up). The sensitivity of the BSA program for prevalent round cases is 85% (age-adjusted) [5]*.* The potential impact of the incorporation of AI into practice was also investigated.

## Materials and methods

This was a retrospective analysis of consecutive prevalent round screening mammograms conducted in 2017 from the Monash BreastScreen service in the state of Victoria.

### Participants

The BSA program is a national population-based program, commenced in 1991, that sets, and annually reviews, performance standards for individual screening services and is responsible for the oversight of service audits every 4 years. Women in the target age group (50–74 years), identified from the electoral roll, are routinely invited for a screening mammogram every two years. Although women aged 40–49 years are not invited, they are eligible for screening. For prevalent screens, BSA has a performance benchmark for recall in the target age group of less than 10% [6].

The study participants were women attending for their prevalent mammogram through Monash BreastScreen, a BSA-accredited service in metropolitan Melbourne, screening up to 60,000 women annually. The service operates through eight separate screening clinics and the images are transmitted to a central facility where they are read by members of a team of 16 radiologists. Monash BreastScreen serves a major city and inner regional population. As false positive screens are recognised as a bigger issue for prevalent than incident screening rounds [5], this study focussed on first-round attendances.

### Standard practice

All two-view digital screening mammograms are read independently by two breast specialist radiologists (readers) and each decides to either clear or recall the case. If recalled, the radiologist scores the case as 3 (equivocal), 4 (suspicious), or 5 (malignant) [7]. Discordant results between the two readers are arbitrated by a third highly-experienced reader. Women with a score of ≥ 3 after arbitration are recalled to a single assessment clinic and those cleared return to routine screening.

Pathology specimens from the assessment clinic are analysed by Monash Health expert breast pathologists (National Association of Testing Authorities accredited). These results are correlated with imaging at a weekly clinical multi-disciplinary meeting. The final histopathology outcomes from subsequent breast surgery are entered into the BreastScreen Victoria (BSV) database.

One of the key measures of the performance of a screening program is false negatives. In the case of screening mammography, women who are identified with interval cancers prior to the next screening round or who are diagnosed with cancer in the next screening round are worth assessing as potential false negative cases from the index screening round. Not all such cases will be false negatives as some cancers develop de novo and have had no signs present during the index round, so in order to assess the issue of false negatives, all such cases need careful review. In our study, interval cancers were defined as invasive BCs detected in the 24 months after a negative screening episode [4]. These cancers are detected outside the screening program and reported to the Victorian Cancer Registry (VCR) under legislative guidelines. The VCR collates and subsequently reports these data back to BreastScreen Victoria [4]. All women with a 2017 prevalent screen who were diagnosed with IBC at their first incident round 2 years later were also reviewed.

For the purposes of this study, all data for the interval cancers and cancers diagnosed in the subsequent round were reviewed with the relevant histopathology data provided by BSV.

Consensus review was undertaken by three experienced radiologists (J.E., J.W., and A.L.) who had a combined experience of over 60 years with arbitration and interval cancer review, on full-definition workstations in a two-stage manner as described in other studies [8]. An initial review was done blinded to both the Transpara score and computer-aided detection (CAD) markings. Once consensus had been reached in relation to any abnormalities on the 2017 images, a second review was undertaken with the AI data included to confirm that the expert panel and the AI program were identifying the same mammographic feature. On the basis of this review, there were no mammographic signs present in the 2017 round, they were called true negatives (true intervals), if there were recognisable signs of a relevant abnormality in 2017 they were called false negatives and if there were subtle signs present in 2017, the cases were classified as having “minimal signs.”

### “Ground truth” data set

This data set is considered to be “ground truth” as it includes all prevalent screening outcome data as well as verified 3-year follow-up. Women identified with IBC outside of the BreastScreen program after a minimum of 24 months from the time of their 2017 scan were classified as “lapsed attenders” and not included with those women diagnosed in 2019.

### AI product

Digital mammograms were obtained using Siemens DR, Mammomat and Inspiration, Sectra DR, and Hologic Dimensions units. The AI software used was Transpara (version 1.7.0*)*, a commercially available product that obtained international regulatory approval in 2018 (US Food and Drug Administration, European Co-operation on Accreditation standard and the Australian Therapeutic Goods Administration). ScreenPoint Medical BV provided the Transpara AI software, which was integrated into the BSV service Sectra IDS7 PACS Platform. Transpara 1.7.0 has been trained on over 1 million mammograms from established test sets. The mammograms used in this study were not used in any part of the algorithm training. The software analyses image information only with no input of patient demographics such as age or BC risk factors, nor does the current version have the capacity to compare studies with prior exams. Image analysis uses deep learning convolutional neural networks to detect calcifications [9] and soft tissue lesions [10] and provides an overall score ranging between 1 and 10, representing the BC risk (where 10 represents the highest chance of malignancy) [11]. The scores of 1–10 represent deciles so that ~10% of women are assigned a score in each of the 10 categories. The current analysis is based solely on the overall score of 1–10 and is not a lesion-specific analysis.

### Ethics approval

Ethics approval for this project was gained by Monash Health Human Research Ethics Committee Low Risk Panel Monash Health (Monash Health Ref: RES-20-0000-166L-61177). All women who are screened through BreastScreen sign a form giving permission for their anonymised test results to be used for research purposes.

### Data analysis

The performance of the radiologists and the AI system was assessed in terms of all lesions (IBC and DCIS) identified in 2017, IBCs identified in 2017, interval BCs (false negatives and minimal signs, not true negatives) and IBCs identified in the subsequent screening round (minimal signs but not true negatives). Data are presented as frequencies and percentages.

The parameters reported include sensitivity, specificity, and positive and negative predictive values, including percentages with confidence intervals. Proportions were compared using the chi-squared test or Fisher’s exact test and the associated *p* values provided.

This study was exploratory in nature and the size of the sample used was pragmatic and determined by the feasibility of having the AI program provide scores for prevalent screens in a single calendar year and for follow-up data to be available for both interval cancers and cancers identified in the subsequent screening round.

## Results

### Participants

In 2017, 53,584 women attended Monash BreastScreen and a subset of 7829 mammograms were prevalent screens. Women having their prevalent screen ranged in age from 40 and 85+ years. The proportion in the target age group 50–74 years was 68.4%.

**Fig. 1:**
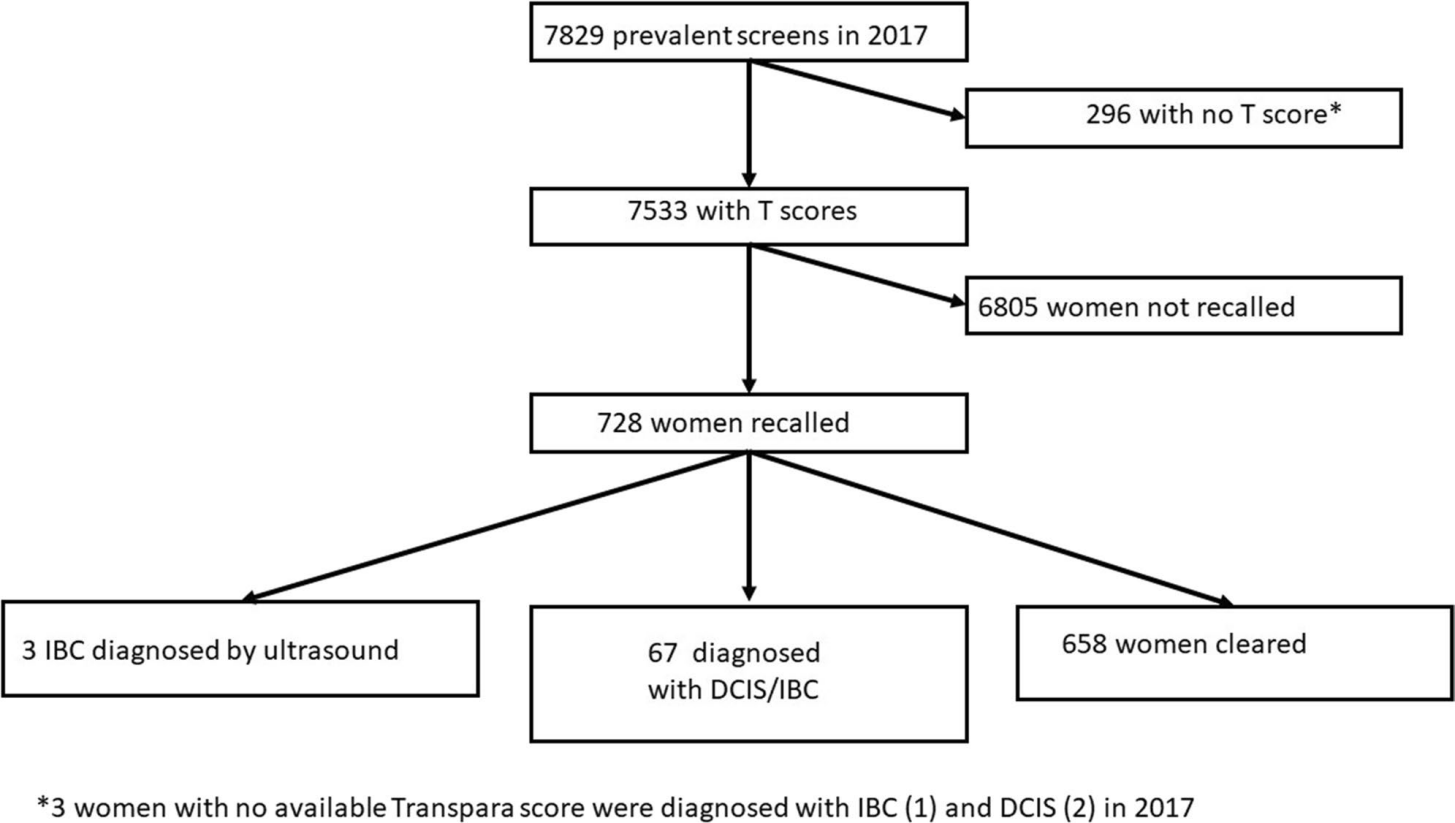
Flow chart showing the number of women with prevalent screens, with Transpara scores available, recalled for further evaluation and diagnosed in the prevalent round

Of the 7829 prevalent screens, 296 were unable to be given a Transpara score for a range of technical reasons, so 7533 (96.2%) women were included in this analysis (Fig. 1). There were 2 cases of DCIS and one case of IBC as well as two interval IBCs within the group of women who did not have a Transpara score.

A total of 728 women from the 7533 (9.66%) were recalled for further assessment with 54 diagnosed with IBC and 13 with DCIS on the basis of mammographic abnormalities. Three women within the recalled group whose cancers were not the recalled abnormality and were diagnosed using ultrasound have been excluded from the analysis as their cancers were mammographically occult (Fig. 1).

A total of 798 women (798/7533 10.6%) received a Transpara score of 10.

**Fig. 2:**
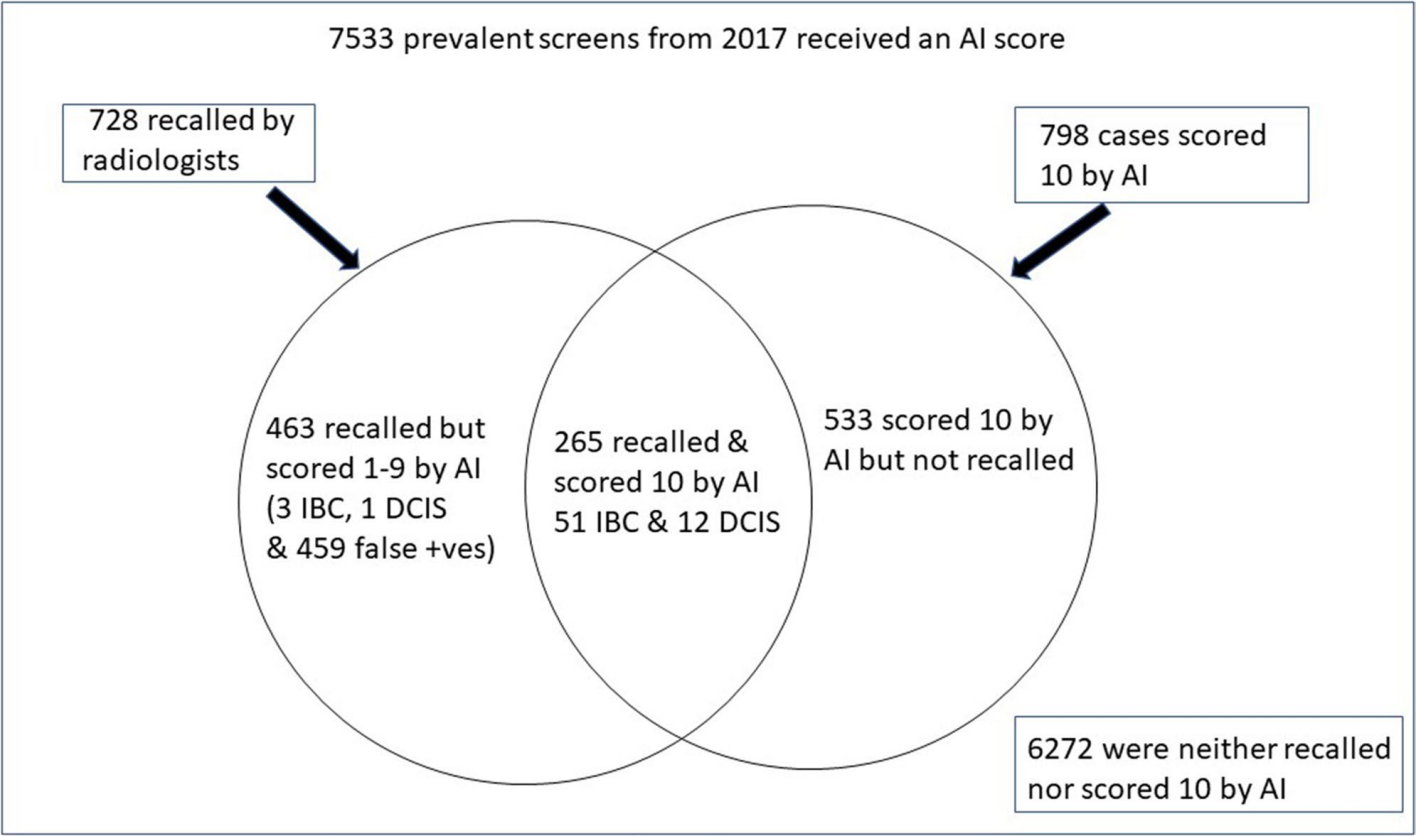
Women recalled to the clinic, women with a Transpara score of 10, and women in both of these groups

Of the 728 women recalled by the radiologists, 36.4% had a score of 10 (Table 1). The overlap between the group of women recalled by the radiologists and the women with a score of 10, along with the lesions diagnosed in 2017, is shown in Fig. 2. There were 265 women who were both recalled and had a score of 10.

**Table 1:** Number, percentage, and cumulative percentage of women recalled to the assessment clinic with Transpara scores 1–10 in the 2017 prevalent round

Transpara score	Number (%)	Cumulative percent
1	4 (0.5)	0.5
2	22 (3.0)	3.6
3	31 (4.3)	7.8
4	27 (3.7)	11.5
5	47 (6.5)	18.0
6	59 (8.1)	26.1
7	60 (8.2)	34.3
8	93 (12.8)	47.1
9	120 (16.5)	63.6
10	265 (36.4)	100.0
Total	728 (100.0)

**Fig. 3:**
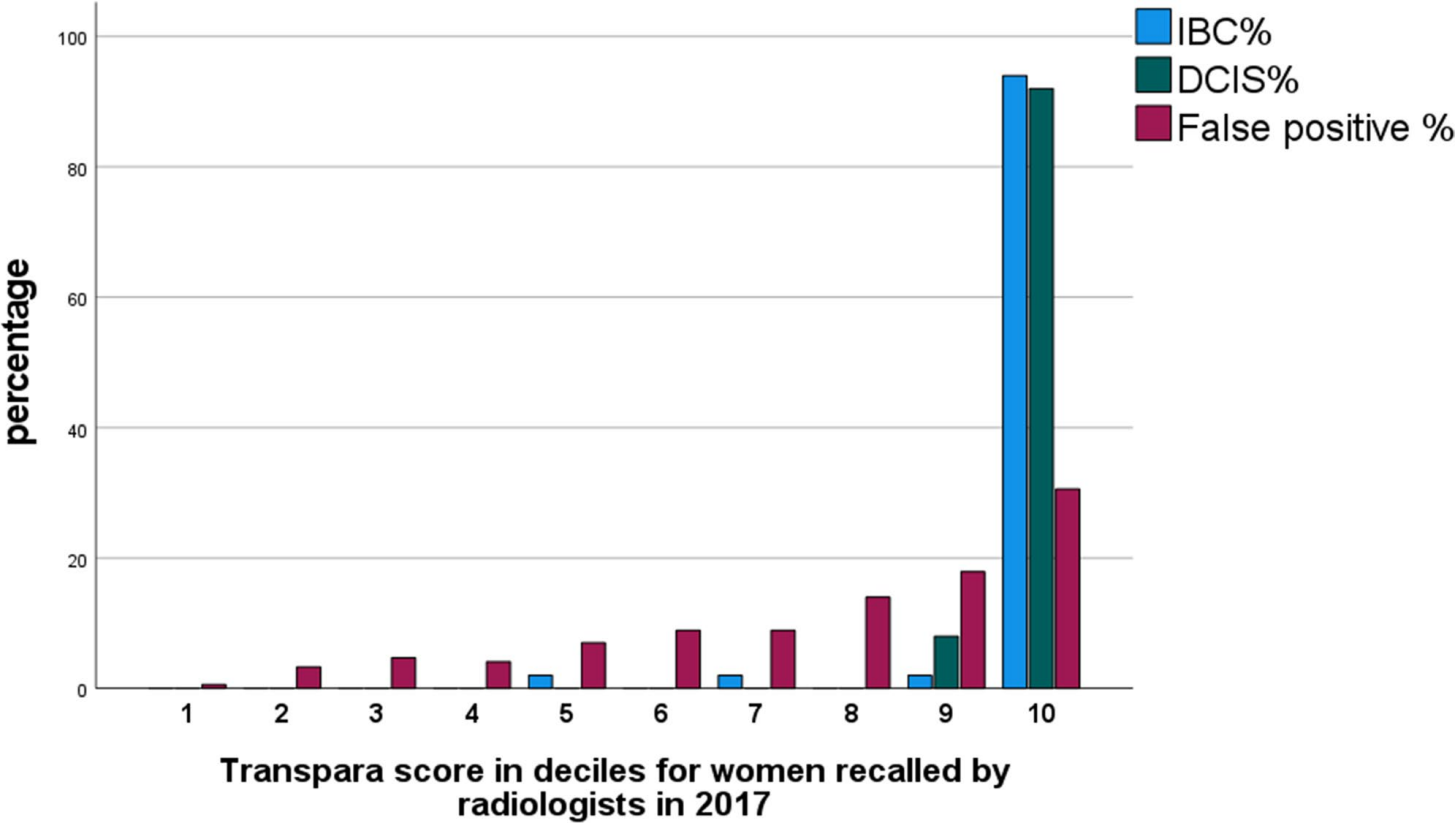
The distribution of women diagnosed with IBC, DCIS, or identified as false positives in the prevalent round of screening (by percentage)

**Fig. 4:**
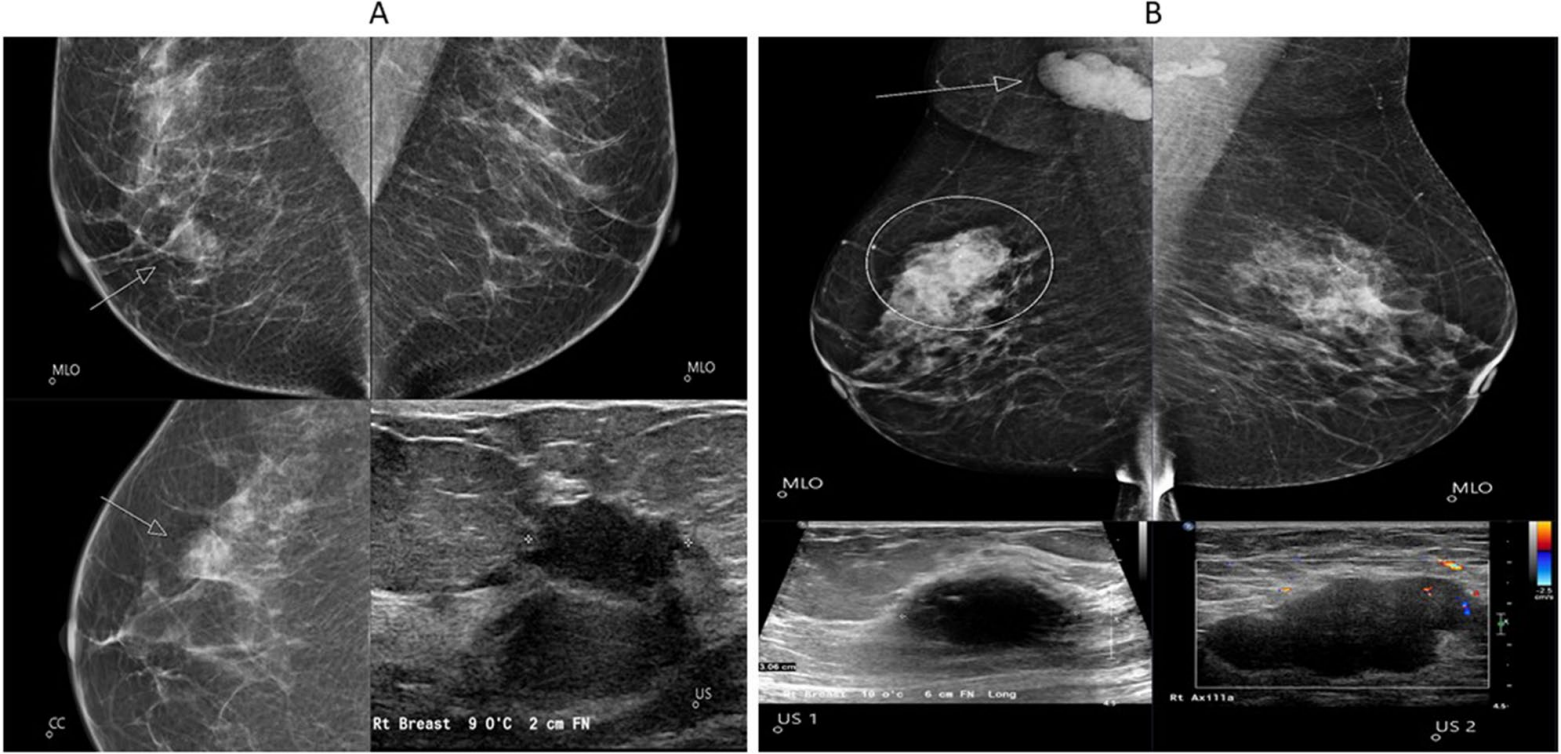
Examples of cancers missed by the AI system. **A** A 15-mm-large invasive ductal cancer (grade 2) with a soft density appearance (arrows) in both views lacks distinct margins. AI risk score of 5/10 and detected by one of two radiologists. MLO, mediolateral oblique view; CC, craniocaudal view, and ultrasound detail. **B** A 29-mm-large invasive ductal cancer (grade 3) (US1) is masked within the dense asymmetric fibroglandular tissue in the right upper outer quadrant (circle) with significant axillary lymphadenopathy (arrow and US 2). Both radiologists recalled this case—AI algorithm fails to recognise the context of the lymph node and provides low-risk score of 7. MLO, mediolateral oblique view; CC, craniocaudal view and US, ultrasound details

Sixty-three of the 67 lesions diagnosed at the 2017 screening (51 IBCs and 12 DCIS) round were within the 265 women who were both recalled by the radiologists and scored a 10. The other 4 lesions (3 IBC and 1 DCIS) were within the group recalled by the radiologists but did not score a 10 (Table 2 and Fig. 3). This represents a sensitivity of 63/67 (94%) for a score of 10. Two of the four cases not classified as high risk by AI are shown in Fig. 4.

**Table 2:** The distribution of Transpara scores in women recalled, diagnosed with IBC or DCIS, false positives and women not recalled, in the 2017 round of screening (number and % by column)

Score	Recalled *n* (%)				Not recalled (%)	Total screened *n* (%)
		IBC^a^ 2017 *n* (%)	DCIS^b^ 2017 *n* (%)	False positives *n* (%)		
1-9	463 (63.6)	3 (5.6)	1 (7.7)	459 (69.4)	6272 (92.2)	6735 (89.4)
10	265 (36.4)	51 (94.4)	12 (92.3)	202 (30.6)	533 (7.8)	798 (10.6)
Total	728	54	13	661	6805	7533

^a^*IBC* invasive breast cancer

^b^*DCIS* ductal carcinoma in situ

From this 2017 cohort, there were 12 interval IBCs diagnosed and 16 IBCs diagnosed in the incident round of 2019. There were no cases of interval DCIS or DCIS in 2019 in women cleared by the radiologists in 2017. A further three women were identified with IBC outside of the BS program, after 24 months, and were classified as “lapsed attenders.” One of these three women had a score of 9 on her 2017 screen.

**Fig. 5:**
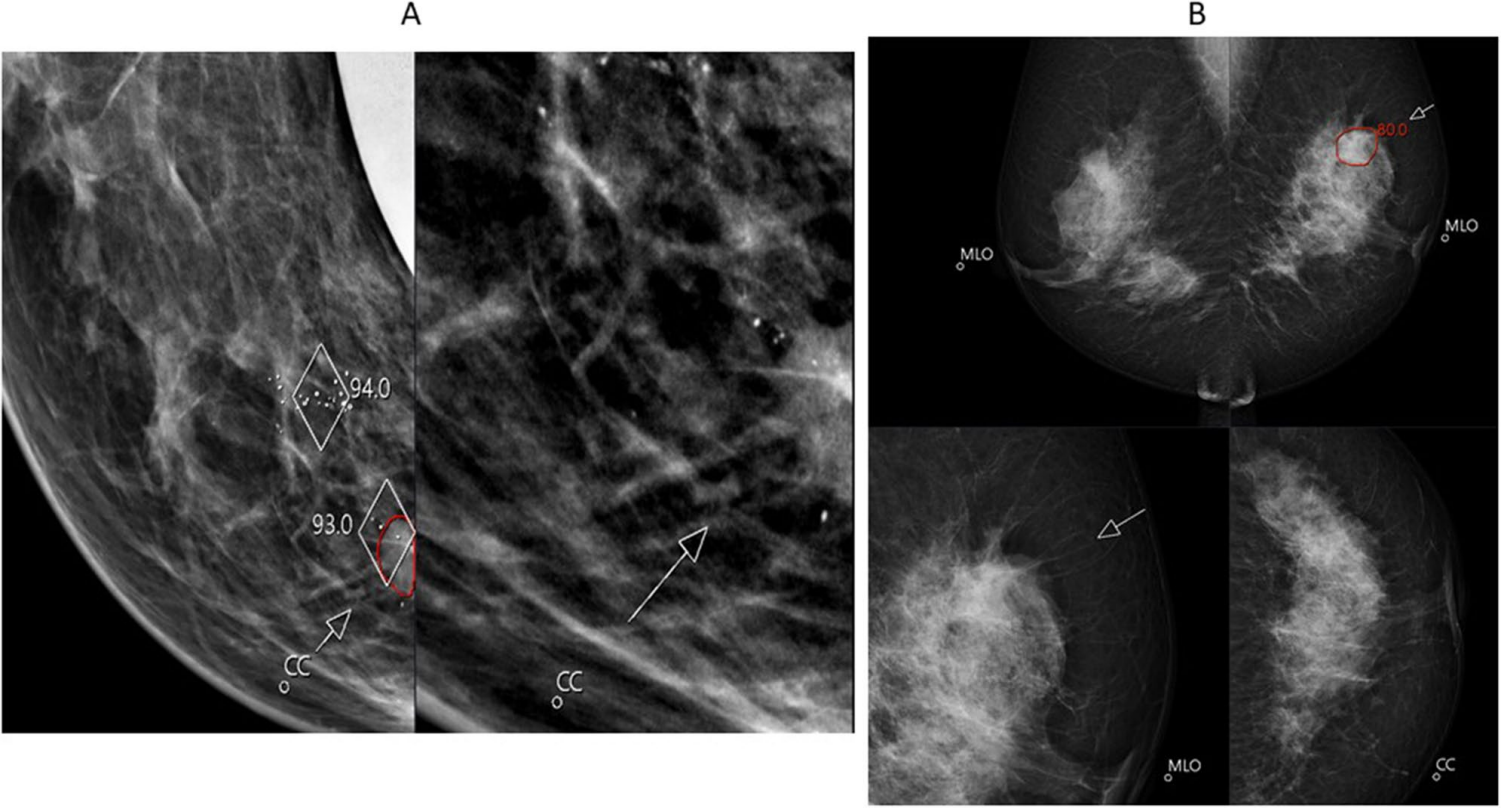
Examples of false negative interval cancers detected by AI. **A** Author’s arrow left CC detail image of a 10-mm-large soft tissue mass in a postero-medial location (red circle CAD marking)—in one view only, scored 10/10 by AI. Adjacent microcalcifications (white diamond CAD markings) attributed a high score also. Clinical presentation 11 months later with a 25 mm invasive ductal cancer (grade 2) on histopathology. CC, craniocaudal view. **B** In the upper left MLO the red circle CAD marking—in one view only—identifies the findings of an asymmetric density with associated architectural distortion of the adjacent glandular tissue (author’s arrows), scored 10/10 by AI. Clinical presentation 7 months later with a 70 mm invasive lobular cancer (grade 2) on histopathology. MLO, mediolateral oblique view CC, craniocaudal view

Of the 12 interval cancers, 5 were considered false negatives, and all 5 of these intervals scored a 10 in 2017 (images from 2 of the 5 are shown in Fig. 5). Four of the 12 intervals were classified as “minimal signs” in 2017 and of these, 2 scored a 10 and 2 scored 1–9 (Table 3). Four of the 16 IBCs diagnosed in 2019 were considered to have had minimal signs in 2017 and one of these four scored a 10 in 2017.

**Table 3:** Interval invasive breast cancers and invasive breast cancers diagnosed in 2019 in relation to the Transpara score in 2017

Transpara score	Interval Total				2019 round		
		Interval False negative	Interval Minimal signs	Interval True negative		No signs in 2017	Minimal Signs in 2017
1–9	5	0	2	3	14	11	3
10	7	5	2	0	2	1	1
Total	12	5	4	3	16	12	4

The performance of the radiologists and the AI program in relation to 2017 diagnoses, interval cancers (excluding true negatives), and 2019 cancers (excluding those with no signs in 2017) is shown in Table 4. The AI program missed 4 lesions detected by the radiologists in 2017 but the AI program identified as high risk some IBCs that later presented as either interval cancers or IBCs in the 2019 screening round. Despite this, across all the comparisons (IBC and DCIS in 2017; IBC only in 2017; IBC in 2017 + interval false negatives + interval minimal signs; IBC in 2017+ interval false negatives + interval minimal signs + 2019 minimal signs) the differences in the performance of the radiologists and the AI program in terms of sensitivity, specificity, positive and negative predictive value were small, or had wide confidence intervals. The details of the invasive cancers detected by the radiologists and/or scored 10 by AI in the 2017 prevalent round are provided in Table 5.

**Table 4:** Performance of the radiologists and Transpara in relation to lesions identified in 2017, then combined with selected interval IBCs and IBCs identified in 2019

	Prevalence	Recall rate	Sensitivity	Specificity	PPV	NPV
2017 IBCs^a^ + DCIS^b^	67/7533 0.89% (0.69–1.13)					
Radiologists		728/7533 9.66% (9.01–10.35)	67/67 100% (94.6–100) *	6805/7466 91.15% (90.48–91.78)	67/728# 9.20 (7.20–11.54)	6805/6805 100% (99.95–100) *
Transpara score 10		798/7533 10.59% (9.91–11.31)	63/67 94.03% (85.4–98.4)	6731/7466 90.16% (89.5−90.82)	63/798 7.89% (6.12–9.99)	6731/6735 99.94% (99.85–99.98)
Diff between proportions *p* value		0.93% (−0.03 to 1.89) *p *= 0.06	5.97% (−0.6 to 14.4) *p *= 0.05	0.99% (0.06 to 1.92) *p *= 0.04	1.31% (−1.50 to 4.17) *p *= 0.36	0.06% (−0.01 to 0.15) *p *= 0.05
2017 IBC^a^ only	54/7533 0.72% (0.54-0.93)					
Radiologists		728/7533 9.66% (9.01–10.35)	54/54 100% (93.4–100) *	6805/7479 90.99% (90.32–91.63)	54/728 7.42% (5.62–9.57)	6805/6805 100.0% (99.95–100.0) *
Transpara score 10		798/ 7533 10.59% (9.91–11.31)	51/54 94.44% (88.33–100)	6732/7479 90.01% (89.33–90.69)	51/798 6.39% (4.69–8.09)	6732/6735 99.96% (99.91–100)
Diff between proportions *p* value		0.93% (−0.03 to 1.89) *p *= 0.06	5.56% (−2.09 to 15.11) *p *= 0.08	0.98% (0.04 to 1.92) *p *= 0.04	1.03% (−1.52 to 3.64) *p *= 0.43	0.04% (−0.02 to 0.12) *p *= 0.10
2017 IBCs^a^ + interval false negatives and intervals minimal signs	63/7533 0.84% (0.64–1.07)					
Radiologists		728/7533 9.66% (9.01–10.35)	54/63 85.71% (74.61–93.25)	6796/7470 90.98% (90.31–91.62)	54/728 7.42% (5.62–9.57)	6796/6805 99.87% (99.75–99.94)
Transpara score 10		798/ 7533 10.59%) (9.91–11.31)	58/63 92.06% (85.38–98.74)	6730/7470 90.09% (89.41–90.77)	58/798 7.27% (5.47–9.07)	6730/6735 99.93% (99.87–99.99)
Diff between proportions *p* value		0.93% (−0.03 to 1.89) *p *= 0.06	6.35% (−5.05 to 17.95) *p *= 0.26	0.89% (−0.05 to 1.83) *p *= 0.06	0.15% (−2.48 to 2.82) *p *= 0.91	0.06% (−0.06 to 0.19) *p *= 0.27
2017 IBCs^a^ + interval false negatives and intervals minimal signs + 2019 IBCs^a^ with minimal signs	67/7533 0.89% (0.69–1.13)					
Radiologists		728/7533 9.66 (9.01–10.35)	54/67 80.60% (69.11–89.24)	6792/7466 90.97% (90.30–91.61)	54/728 7.42% (5.62–9.57)	6792/6805 99.81% (99.67–99.90)
Transpara score 10		798/ 7533 10.59% (9.91–11.31)	59/67 88.06% (80.30–95.82)	6727/7466 90.10% (89.42–90.78)	59/798 7.39% (5.57–9.21)	6727/6735 99.88% (99.80–99.96)
Diff between proportions *p* value		0.93% (−0.03 to 1.89) *p *= 0.06	7.46% (−5.07 to 19.89) *p *= 0.24	0.87% (−0.07 to 1.81) *p *= 0.07	0.03% (−2.61 to 2.71) *p *= 0.98	0.07% (−0.07 to 0.22) *p *= 0.30

^*a*^*IBC* invasive breast cancer; ^b^*DCIS* ductal carcinoma in situ; *PPV* positive predictive value; *NPV* negative predictive value; Numbers in brackets represent the 95% confidence intervals for the percentages

^*^One-sided confidence interval; Chi-square or Fisher’s exact test as appropriate

*p* values are for between-group comparisons (radiologists versus Transpara score of 10) for each outcome

**Table 5:** Details of 2017 prevalent invasive cancers and interval cancers (false negative and minimal signs)

Detected by	Number of women	Histology and grade ^a^	Size range (mm)	Node positive	ER or PR positive	Triple-negative status
AI score 10 and radiologist	51					
	8	Invasive ductal Grade 3	14–43 median 26	4	5	3
	21	Invasive ductal Grade 2	1.5–41 median 20	6	21	0
	13	Invasive ductal Grade 1	5–26 median 12	1	13	0
	3	Invasive ductal Grade unknown^b^	0.4–0.8	0	2	1
	1	Histology and Grade unknown	21	0	1	0
	3	Invasive lobular Grade 2	11–30	1	3	0
	1	Mixed ductal /lobular Grade 3	16	0	1	0
	1	Mucinous (colloid) invasive Grade 1	25	0	1	0
AI score < 10 and radiologist	3					
	1	Invasive ductal Grade 3	29	1	1	0
	2	Invasive ductal Grade 2	10–15	0	2	0
AI score 10-Interval cancers	7					
False negatives	5					
	3	Invasive ductal Grade 3	21–34	3	N/A	
	1	Invasive ductal Grade 2	25	0	N/A	
	1	Invasive lobular Grade 2	70	1	N/A	
Minimal signs	2	Invasive ductal Grade 3	13 and one multifocal	0	N/A	

^a^ Grade 1 well differentiated; Grade 2 Intermediate differentiation; Grade 3 poorly differentiated

^b^ Detected by recalled microcalcifications (DCIS); the lesion is categorised by its < 1 mm invasive component

N/A not available

## Discussion

Our study is an independent (not industry-led) assessment of the performance of an established AI program in the prevalent round of screening in a “work as usual” accredited screening mammography program where the likelihood of a true positive lesion is up to 1.2% [4], and where data on interval cancers and next round cancers were available (ground truth) [12]. Our focus on a prevalent screening round was deliberate as recall rates are consistently higher in prevalent than incident rounds [5] and the high negative predictive value for AI demonstrated in this study has the potential to reduce unnecessary recalls (false positives) in this group (Table 2 and Fig. 3).

A Transpara score of 10 identified 63/67 cases of IBC or DCIS in the 2017 screening round, however, a Transpara score of 10 did identify some interval IBCs and IBCs identified in the subsequent screening round, although the difference in the sensitivity between the AI score and the radiologists was not different statistically. A large review of interval cancers [13] noted the problem of small invasive tumours “masked” by dense fibroglandular tissue or with “minimal signs.” Typically, 20–25% of intervals were classified as false negatives, where observable mammographic features were missed by radiologists. In our study, 5/12 intervals were considered as false negatives at “blinded” expert review, and all scored 10 (highest risk) by Transpara and marked by the AI algorithm (images 5A and 5B). Our study confirms the role of AI in the minimisation of “false negatives” [14, 15]. It was notable that two of the four women who were missed by AI in the prevalent round had signs that were not missed by a radiologist [16], demonstrating the need for all images to be read by at least one radiologist [12]. We consider that commencing the task of integrating AI into screening mammography and upskilling radiologists to work in this setting is better started from a position supporting “human in the loop” collaboration [17]. Unlike some recent authors, we would not advocate for a scheme where some images are only analysed by AI and not read by a radiologist [14, 16, 18]. We envisage a system similar to that of McKinney [15] and Raya-Povedano [19]. An iterative review of reader performance will be required to avoid increasing the recall rate from the “cancer-enriched” groups [1]. A review of AI errors is also important [12]. Radiologists also need to understand the psychology of how AI affects their reporting [12, 18, 20]. Senior clinician oversight will be pivotal as protocols evolve which must achieve acceptable clinical standards both for organisations responsible for the governance of breast screening, as well as for the women being screened [12, 20–22]. The incorporation of AI into the reading of screening mammograms has been shown to reduce radiologist workload in some settings [23] although this is not universally the case [22]. Resources saved by no longer having all images read by two independent radiologists could be invested in optimising case review and arbitration.

A strength of this study is that the mammograms analysed represent a consecutive series in one calendar year from one screening service and the outcomes include IBCs and DCIS diagnosed in the prevalent round, as well as 3-year follow-up. Limitations included that a small proportion of mammograms could not be scored by the AI program for technical reasons and this would need to be addressed if AI was to be introduced into routine practice. Furthermore, the study was limited to prevalent round screens from a single calendar year.

## Conclusion

Our study has shown that the AI program used in our study has a similar sensitivity to that of expert radiologists in the prevalent round, could reduce interval cancers (false negatives), and has a high negative predictive value for scores 1–9 demonstrating its potential role in false positive reduction.

## Abbreviations

AI: Artificial intelligence
BC: Breast cancer
BSA: BreastScreen Australia
BSV: BreastScreen Victoria
DCIS: Ductal carcinoma in situ
DR: Digital radiography
IBC: Invasive breast cancer
PACS: Picture archival and communications system
VCR: Victorian Cancer Registry

## Declarations

### Guarantor

The scientific guarantor of this publication is Prof Robin Bell.

### Statistics and biometry

One of the authors (RJB) has significant statistical expertise.

### Study subjects or cohorts overlap

No AI data on the study subjects have been previously reported.

### Methodology

• retrospective

• observational

• performed at one institution
